# Structural Analysis of the Effect of Asn107Ser Mutation on Alg13 Activity and Alg13-Alg14 Complex Formation and Expanding the Phenotypic Variability of ALG13-CDG

**DOI:** 10.3390/biom12030398

**Published:** 2022-03-04

**Authors:** Karolina Mitusińska, Artur Góra, Anna Bogdańska, Agnieszka Rożdżyńska-Świątkowska, Anna Tylki-Szymańska, Aleksandra Jezela-Stanek

**Affiliations:** 1 Tunneling Group, Biotechnology Centre, Silesian University of Technology, 44-100 Gliwice, Poland; a.gora@tunnelinggroup.pl; 2 Department of Biochemistry, Radioimmunology and Experimental Medicine, The Children’s Memorial Health Institute, 04-736 Warsaw, Poland; a.bogdanska@ipczd.pl; 3 Anthropology Laboratory, Children’s Memorial Health Institute, 04-736 Warsaw, Poland; a.rozdzynska-swiatkowska@ipczd.pl; 4 Department of Pediatrics, Nutrition and Metabolic Disorders, Children’s Memorial Health Institute, 04-736 Warsaw, Poland; a.tylki@ipczd.pl; 5 Department of Genetics and Clinical Immunology, National Institute of Tuberculosis and Lung Diseases, 01-138 Warsaw, Poland

**Keywords:** ALG13-CDG, c.320A>G variant (p.Asn107Ser), congenital disorder of glycosylation, transferrin isoelectric focusing, human Alg13 modeling

## Abstract

Congenital Disorders of Glycosylation (CDG) are multisystemic metabolic disorders showing highly heterogeneous clinical presentation, molecular etiology, and laboratory results. Here, we present different transferrin isoform patterns (obtained by isoelectric focusing) from three female patients harboring the *ALG13* c.320A>G mutation. Contrary to other known variants of type I CDGs, where transferrin isoelectric focusing revealed notably increased asialo- and disialotransferrin fractions, a normal glycosylation pattern was observed in the probands. To verify this data and give novel insight into this variant, we modeled the human Alg13 protein and analyzed the dynamics of the apo structure and the complex with the UDP-GlcNAc substrate. We also modeled the Alg13-Alg14 heterodimer and ran multiple simulations of the complex in the presence of the substrate. Finally, we proposed a plausible complex formation mechanism.

## 1. Introduction

Congenital Disorders of Glycosylation (CDG) are multisystemic metabolic disorders caused by defects in N-linked, or O-linked oligosaccharides, shared substrates, glycophosphatidylinositol (GPI) anchors, or dolichols pathways [1]. There are two types of CDGs: type I CDGs are diagnosed when there are defects in the assembly or transfer of the dolichol-linked glycan in either the cytosol or the endoplasmic reticulum (ER). Type II CDGs are diagnosed when there are processing defects of the glycan in the ER or the Golgi apparatus.

*ALG13* gene (Asparagine-linked glycosylation homolog 13) dysfunction is related to type I CDGs. The product of the *ALG13* gene, the Alg13 protein, is involved in the N-glycosylation process. Several mutations of the *ALG13* gene have been reported, related mostly to epilepsy [2,3,4,5,6,7], but also to kidney failure [8]. Considering ALG13-CDG, the c.320A>G (p.Asn107Ser) variant is by far the most frequent in affected female heterozygotes [7]. To date, it has been described in 46 females and only three males [2,4,5,6,7,9,10]. Yet, the details of the effect of this mutation on glycosylation process remains unknown.

The Alg13 protein is a highly conserved X-linked uridine diphosphate (UDP)-N-acetylglucosaminyltransferase required for the transfer of N-acetylglucosamine (GlcNAc) onto the extending lipid-linked oligosaccharide (LLO) structure, dolichol-P-P GlcNAc [11]. This early step in the LLO synthesis pathway is crucial for the N-glycosylation process, as was shown by studies on yeasts. The yeast Alg13 protein is localized mostly in the cytoplasm. However, it can be also found in the ER as a part of a heterodimer, while it binds with an ER transmembrane protein Alg14. The ER-bound complex of Alg13-Alg14 is crucial for both cell viability and proper N-glycosylation, since lack of this interaction causes profound defects in both [11]. Other studies showed that the yeast Alg13 and Alg14 proteins are distantly related to the bacterial MurG UDP-GlcNAc glycosyltransferase which is involved in peptidoglycan and are implicated to playing a role in N-glycosylation [12,13].

In early 2005, Chantret et al. [12] suggested that for the second step of N-glycosylation in eukaryotes, two proteins homologous to the N-terminal and C-terminal domains of the bacterial MurG are required. Later that year, Gao et al. confirmed their results, pointing out Alg13 and Alg14 as the proteins involved, and showed that Alg14 functions as a membrane anchor that recruits Alg13 to the cytosolic face of the ER, where catalysis of GlcNAc2-PP-dol occurs [13]. Wang et al. speculated that since the sugar donor binding domain and the sugar acceptor binding domain exist separately in Alg13 and Alg14 in some organisms, their bipartitioning can offer many advantages, such as flexibility in the protein activity regulation and the ability to recruit substrates in both the cytosol and the membrane [14]. They proposed Alg13 as a general UDP-GlcNAc carrier, regulating the delivery of UDP-GlcNAc to a variety of membrane-associated proteins involved in complex carbohydrate synthesis. The modification of the binding affinity could provide vital consequences. Moreover, Wang et al. analyzed the behavior of yeast Alg13 in the presence of the UDP-GlcNAc substrate. They showed that the residues located on the two helices are the most disturbed by the presence of the substrate [14].

Protein N-glycosylation is a two-step process: it starts with the transfer of an oligosaccharide from a lipid-linked oligosaccharide onto proteins and is initiated by UDP-GlcNAc:dolichol phosphate *N*-acetylglucosamine-1-phosphate transferase (GPT) encoded by *DPAGT1*. It was reported that deficiencies of *DPAGT1* are also related to a group of CDG disorders (formerly Ij type) [13]. The second step differs between prokaryotes and eukaryotes. It has been previously shown that prokaryotes require a monomeric enzyme corresponding to MurG, while in the case of eukaryotes the role of bacterial MurG is carried out by two enzymes, such as Alg13 and Alg14.

There is a high sequence similarity between the prokaryotic and eukaryotic enzymes involved in the first step of N-glycosylation [15]. The product of the *DPAGT1* gene, the GPT enzyme, is mostly membrane-bound in the ER. For example, the hamster GPT enzyme, which was examined by Dan et al. [16], consists of 10 membrane units, so-called spans, connected by loops, two of which have a lumenal orientation (loop 6/7 and the C-terminal region), while two others (loops 1/2 and 9/10) are cytosolic. Moreover, loop 9/10 has highly conserved elements that are essential for enzyme function; the C-terminal of the 10 span plays a role in the stabilization of the GPT [17], while spans 2 and 7 contain potential dolichol recognition sequences. Their more recent results suggest that GPT may occur in an oligomeric state [18]. It should be noted here that the GPT protein, a product of the *DPAGT1* gene is called Alg7 in *S. cerevisiae* [16], and its interaction with the Alg13 and Alg14 has already been analyzed by Chantret et al. [12], as well as Noffz et al. [19], who were first to show the Alg7-Alg13-Alg14 interaction in eukaryotes. Moreover, their results suggest that the Alg7-Alg13-Alg14 complex might be present in a multimeric form. Interestingly, they also showed that the Alg7 and Alg13 are not directly in contact, but rather they associate by close contact with Alg14 [19].

Taken together all the above mentioned information, a clear mechanism appears; however, it still requires experimental confirmation. N-glycosylation is a process which takes place on the endoplasmic reticulum, and in the case of eukaryotes requires the presence of three enzymes, Alg7, Alg13, and Alg14, which can create a complex together. They may share a common binding site cavity, or the cavity is separated by some flexible regions, which act as molecular gates [20]. Alg7 is mostly bound in the membrane of the ER, and it facilitates the first step of the process. The Alg14 protein is in close contact with the Alg7. The only soluble protein in this complex, the Alg13, acts as a transporter, delivering the substrate directly into the Alg7-Alg14 complex. Since the investigated mutation is localized on the human Alg13 loop which participates in both Alg13-Alg14 dimer formation and the substrate binding, we hypothesized that it can have multi-level consequences: (i) influence the substrate carrier properties, (ii) modify the activity/selectivity of the Alg13-Alg14 dimer, and finally (iii) disrupt the dimer formation.

Contrary to other known variants related to *ALG13* dysfunctions, where transferrin isoelectric focusing (IEF) revealed notably increased asialo- and disialotransferrin fractions, in the patients harboring the p.Asn107Ser mutation, a normal glycosylation pattern was observed (in four individuals), similarly to mass spectrometry (MS) results, previously described in one individual [2]. There is, however, one report showing MS evidence of the slightly reduced glycosylation with the absence of one glycan [5]. Since the laboratory data are ambiguous, and the number of reported c.320A>G ALG13-CDGs is still very limited, we would like to present different IEF patterns observed in the transferrin IEF results among the female patients described in our previous publication [10], in order to expand the reported phenotypic variability resulting from the c.320A>G *ALG13* variant. We complemented the report by an exhaustive homology modeling study of the human Alg13 protein and the complex formation with Alg14. We ran in total 6 µs of molecular dynamics (MD) simulations to examine the behavior of the Alg13 protein, its complex with the UDP-GlcNAc substrate and the Alg13-Alg14 heterodimer in the presence of the substrate. We identified a conformational change which is crucial for the substrate transport and heterodimer formation that was not observed in the Asn107Ser variant of the human Alg13 protein. We hope that our results shed light on the role of the human Alg13 protein.

## 2. Materials and Methods

### 2.1. Patients

#### 2.1.1. General Information

Three female patients with a de novo c.320A>G variant in the *ALG13* gene were described previously by Paprocka et al. [10]. In all patients, genetic testing (exome sequencing in Patients 1 and 2, and an epilepsy genes panel in Patient 3) was performed due to the observed developmental delay, accompanied by epilepsy.

#### 2.1.2. Anthropometry

Anthropometric measurements were undertaken for Patients 1 and 2. A Wolański liberometer (a type of an infantometer accurate to 1 mm) was used to measure the supine length of Patient 1. A stadiometer (accurate to 1 mm) was used to measure the standing height of Patient 2. Their weight was determined using an electronic scale accurate to 0.05 kg. A non-stretchable tape was used to measure their head and chest circumferences (accurate to 5 mm). Anthropometric measurements were obtained for each patient (Supplementary Table S1) following standard anthropometric techniques [21,22]. Measurements were recorded to the nearest millimeter using standard calibrated equipment: GPM sliding and spreading, blunt-ended calipers, and an anthropometer. The following parameters were obtained: the somatometric and craniofacial widths, lengths, depths, and heights, as well as details of the eye, nose, and mouth structures.

The age and sex data were standardized based on the mean and standard deviation of the examined feature in a given age group of the healthy children using Polish reference charts [23,24,25].

#### 2.1.3. IEF

The transferrin isoforms were examined by IEF in agarose gel on a Multiphore 2117 apparatus (LKB). The serum transferrin was saturated with iron by the addition of ferric citrate and sodium bicarbonate. The iron-saturated transferrin was electrophoretically separated into fractions using a pH gradient of 5–7 formed by the ampholines added to the 1% agarose gel. The position of the isoforms in the gel depended on the amount of electronegative sialic acid residues on the glycans associated with each isoform. After separation, the isoforms were precipitated into the gel with specific polyclonal-rabbit anti-human transferrin antibodies. Proteins that were not bound by antibodies were washed away from the gel. The gel was dried, and the protein complexes were stained within a 0.5% Coomassie Brilliant Blue solution. The percentage of individual fractions were determined using an Appraise densitometer (Beckman). The procedure was based on the method described by Van Eijk et al. [26] with our modification [27].

#### 2.1.4. Capillary Electrophoresis (CE)

CE was used for detection of CDT—Carbohydrate Deficient Transferrin (asialo-, monosialo- and disialotranferrin) [28]. The CAPILLARYS System (Sebia) with the CAPILLARYS CDT kit, and the MINICAP system were used. The MINICAP system uses the principle of electrophoresis in a thin capillary in an electrolyte solution. Charged molecules were separated on the basis of their electrophoretic mobility in an alkaline buffer at a defined pH of 8.8 using high-voltage direct current [29].

### 2.2. Protein Modeling

#### 2.2.1. Homology Modeling

The Alg13 homology model was created by the I-TASSER webserver [30] using the default settings. The web server provided five different homology models, and the best model was selected based on the TM-score [31]. We modeled the short *ALG13*-is2 isoform of human Alg13 protein (UniProt ID: Q9NP73).

The selected models of the Alg13 protein and the Alg14 structure downloaded from the SWISS-MODEL repository (ID: Q96F25) [32] were used to build a homology model of an Alg13-Alg14 complex using the *Escherichia coli* MurG in a complex with UDP-GlcNAc (PDB ID: 1nlm) and the *Pseudomonas aeruginosa* MurG structure in complex with UDP-GlcNAc as templates. 10,000 homology models were created using the automodel class implemented in Modeller 9v19 [33]. The substrate position was modeled according to the *P. aeruginosa* MurG structure, and the best model was selected based on the lowest DOPE score.

The quality of all used models, namely the Alg13 homology model, the Alg14 structure, and the dimer model were evaluated by ProSA [34] and PROCHECK [35]. In general, the lower values represent the more native-like models.

#### 2.2.2. MD Simulations

The H++ server [36] was used to protonate used models using standard parameters and pH of 6.8. The UDP-GlcNAc substrate structure (ChemSpider ID: 393240) was parameterized using the AMBER 18 antechamber package. Water molecules were placed using a combination of 3D-RISM theory [37] and the Placevent algorithm [38]. The AMBER 18 LEaP package [39] was used to immerse the models in a truncated octahedral box with a 10 Å radius of TIP3P water molecules and prepare the systems for simulation using the ff14SB force field [40]. The PMEMD CUDA package of AMBER 18 software was used to run the molecular dynamics simulations. The minimization procedure consisted of 2000 steps, involving 1000 steepest descent steps, followed by 1000 steps of conjugate gradient energy minimization, with decreasing constraints on the protein backbone (500, 125, and 25 kcal × mol^−1^ × Å^−2^), and a final minimization with no constraints of conjugate gradient energy minimization. Next, a gradual heating was performed from 0 K to 300 K over 20 ps using a Langevin thermostat with a collision frequency of 1.0 ps^−1^ in periodic boundary conditions with constant volume. The equilibration stage was run using the periodic boundary conditions with a constant pressure for 1 ns with 1 fs step using Langevin dynamics with a collision frequency of 1 ps^−1^ to maintain the temperature. The production stage was run for 200 ns in five repetitions for all models, with a 2 fs time step using Langevin dynamics with a collision frequency of 1 ps^−1^ to maintain a constant temperature. The long-range electrostatic interactions were modeled using the particle mesh Ewald method with a non-bonded cut-off of 10 Å, and the SHAKE algorithm was used to maintain the geometrical constraints. The coordinates were saved at an interval of 1 ps.

#### 2.2.3. Assessment of the Stability of the Alg13-Alg14 Complex with the UDP-GlcNAc Substrate

The MD simulations were processed with the Molecular Mechanics Generalized Born Surface Area (MMGBSA) and the Molecular Mechanics Poisson-Boltzmann Surface Area (MMPBSA) implemented in AMBER 18 suite [39] to obtain binding free energies for the Alg13-Alg14 complex with the substrate. Every 50th frame from the last 10 ns of the simulation was selected to be processed by the routine and the final energy was averaged from the five repetitions. The per-residue free energy decomposition was also calculated for the same set of frames.

#### 2.2.4. Introduction of Mutations in Position 107

FoldX suite [41] was used to introduce the substitutions of the Asn residue in position 107. FoldX introduces the substitution(s) of the selected amino acid(s), optimizes the structure of a new variant, and calculates the difference in the Gibbs free energy of protein folding between the native protein and the mutant variants in kcal/mol. The lower the difference in energy, the more stable the mutant variant should be.

#### 2.2.5. Assessment of the Effect of the Introduced Mutation

The PredictSNP [42] server was used to assess the effect of the Asn107Ser mutation. The PredictSNP server contains the results of eight different tools: MAPP, PhD-SNP, PolyPhen-1, PolyPhen-2, SIFT, SNAP, nsSNPAnalyzer, and PANTHER. Each tool predicts the mutation results in terms of neutral or deleterious effects as well as provides a percentage of confidence of its predictions.

## 3. Results

This section is divided into experimental and computational parts. The first part presents the transferrin isoform patterns of our three patients obtained by IEF. We observed differences in the transferrin isoform patterns between our patients, as well as between samples taken from the same patient at different times. These observations have led us to search for differences in the mechanism of action between the native and Asn107Ser variants of the Alg13 protein. Therefore, the experimental part is complemented by the computational part. The latter describes the homology modeling of human Alg13 protein, the molecular dynamics simulations of the apo (“empty”) structure of Alg13, and its complex with the UDP-GlcNAc substrate. We also created an Alg13-Alg14 complex homology model based on bacterial MurG proteins with a bound substrate, and finally we ran MD simulations of the Alg13-Alg14 heterodimer in the presence of the substrate.

### 3.1. Clinical Data

For detailed descriptions of the patients, please refer to our previous article by Paprocka et al. [10]. Here we present the anthropometric results and the transferrin IEF data, as well as a clinical update.

During the latest assessment, Patient 1 was 3 years and 8 months of age. She attended a kindergarten for children with special needs, to which she adapted very well. She needed foot orthoses, but was able to walk independently with some coordination issues. She has a broad-based gait with uplifted forearms. Her speech was still severely delayed, she could say only “mama”. Seizures were not observed and antiepileptic drugs had been reduced.

Patient 2, now 6 years old, started to walk independently at the age of four, and it is no longer a wide-based gait but more natural. She is continuously rehabilitated and undergoes special education with a psychologist, neurologist, SI therapist, hand therapy, and group social therapy. There are still problems with eating—she often chokes, does not eat on her own (cannot use a spoon/fork), and cannot drink from a cup despite constant training at home and in therapy classes—she drinks from a bottle. The patient still does not speak, and her imitation ability is very limited. For two years, she has had alternative communication introduced. When asked, she can often show the correct answer (but only those she is regularly shown and told); she distinguishes between symbols yes/no and often shows adequately to the situation. Unfortunately, the proband understands very little, can only react to her name, and addresses regularly repeated requests such as “hands up”. She does not report basic physiological needs and is still diapered. As for the patient’s medications, she is still on Sabril and Lamitrin S (for which the dosage is increasing, trends toward monotherapy). Epileptic seizures occur only during sleep, but they are quite frequent—every second/third night. They appear as follows: the patient wakes up during the night, and for a few minutes, she periodically blinks her eyes and tenses her body, after which she falls asleep. Seizures very rarely require Relsed infusion. The patient has been attending a private kindergarten for the second year now. Although the progress is slow, she is very eager to participate in the classes and even demands continuing when they are over. The patient has a very gentle and cheerful disposition, smiles often and loves to cuddle. She is very friendly; she demonstrates many behaviors on the autism spectrum, but the diagnosis has not been made.

Patient 3, aged 7 years and 9 months, is the most severely delayed. She is wheelchaired, cannot speak and suffers from everyday epileptic attacks, especially during bedtime. As the patient cannot swallow solid pieces, she only eats liquid food. Her diet is high in carbohydrates, mainly potatoes, and therefore a ketogenic diet is not possible.

### 3.2. Anthropometric Measurements

**Figure 1:**
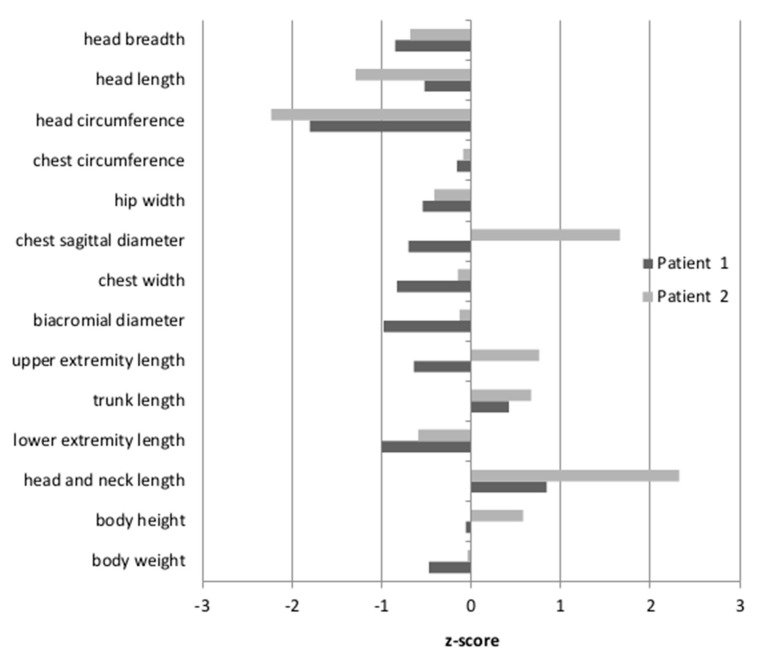
Z-scores for Patients 1 and 2. The results of anthropometric measurements were standardized, based on the mean and the standard deviation of the examined feature in a given age group of the healthy Polish children.

Anthropometric measures were undertaken for Patients 1 and 2, while Patient 3 was unavailable. The results were then standardized, based on the mean and the standard deviation of the examined feature in a given age group of healthy Polish children. The resulting z-scores for Patient 1 and Patient 2 are shown in Figure 1. The body height of the patients was between the 50th and 75th percentile, therefore within the normal range (z-score −0.06–0.58). For Patient 2, the length of the head and neck were above the 97th percentile (z-score 2.32). For Patient 1, only the tendency for an elongated head and neck was observed, although the measured values fell within the normal range (z-score 0.84). Moreover, only Patient 2 had a convex chest (the z-score for the sagittal chest diameter was 1.66). The head circumferences in both patients were below the 3rd percentile (Patient 1: z-score −1.8; Patient 2: z-score −2.23). Other body proportion parameters of both patients did not differ from their healthy peers.

### 3.3. Transferrin Isoforms Pattern Detection by IEF

**Figure 2:**
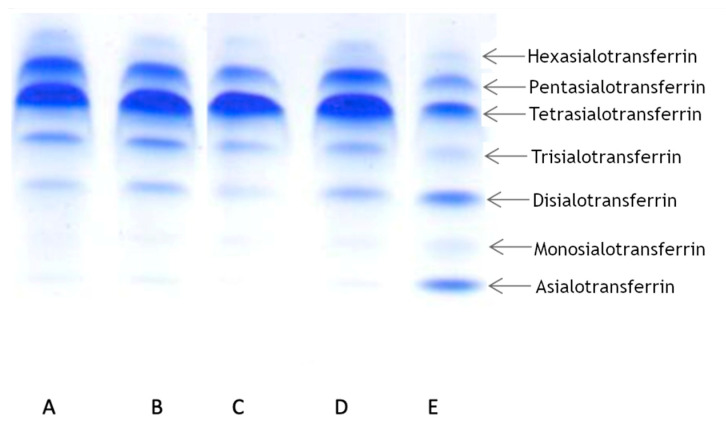
Isoelectric focusing (IEF) of transferrin in patients with *ALG13* c.320A>G variant. (**A**) Patient 3: normal profile; (**B**) Patient 3 (another sample): slight increase of disialotransferrin; (**C**) Patient 1: normal profile; (**D**) Patient 2: mild pattern of type I; (**E**) Positive control (CDG type I).

To determine the transferrin pattern, we used IEF. For each patient we ran two analyses at different times. Patient 1 showed a normal transferrin pattern in both trials. Patients 2 and 3 displayed abnormalities in at least one trial. Patient 2 displayed a slight elevation of asialo-, monosialo-, and disialo fraction in the first trial, while in the second trial the pattern was normal. Patient 3 displayed a normal pattern in the first trial, but in the second trial we observed a slight elevation in only the disialo- fraction. The results are summarized in Table 1 and presented in Figure 2.

In light of the available publications on the subject, these results seemed surprising. Therefore, we also analyzed samples using another method (CE), which confirmed the results (Table 2). Patient 1 displayed normal value of transferrin level, while two other patients displayed increased levels of transferrin. These findings encouraged us to examine the mechanism of Alg13 protein dysfunction, so we used an in silico approach to determine the structure of human Alg13 and the Alg13-Alg14 heterodimer.

### 3.4. Alg13 Protein Modeling

**Figure 3:**
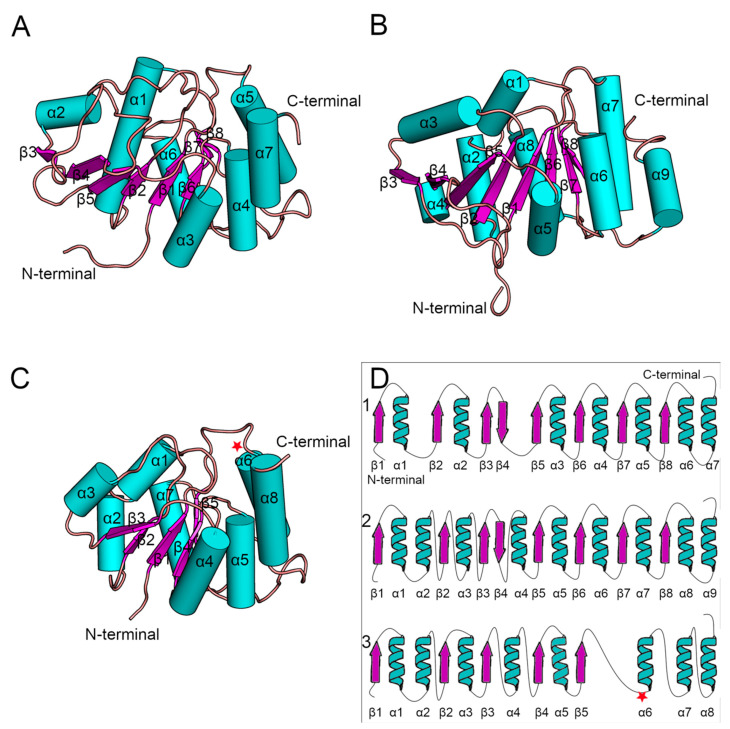
Comparison of known structures of the yeast (*Saccharomyces cerevisiae*) Alg13 structures with the homology model of human Alg13 (hAlg13_hm). (**A**) The yeast Alg13 structure solved by Wang et al. [14] (PDB ID: 2jzc), (**B**) the yeast Alg13 structure solved by Raman et al. [43] (PDB ID: 2ks6), (**C**) the homology model of the human Alg13 structure, (**D**) schematic representation of the Alg13 structures with corresponding secondary structures positioned in the same column: (1) Wang’s model, (2) Raman’s model and (3) hAlg13_hm homology model. The residue on the 107 position of the hAlg13_hm is marked by a red star.

Since the crystal structure of human Alg13 protein remains unknown, we created a homology model using the I-TASSER webserver, which we will refer to as hAlg13_hm. The model had a structural similarity with the yeast Alg13 structures described by Wang et al. [14] and Raman et al. [43], despite relatively low sequence similarity (28%; human Alg13 protein UniProt ID: Q9NP73). The model was evaluated by ProSA and PROCHECK to assess its overall quality. Both tools identified that the model has good quality (ProSA Z-score: −7.62, PROCHECK overall score: −0.58). It is worth noting that the sequence of human Alg13 protein was also shorter (165 aa) relative to the yeast Alg13 (224 aa in Wang’s model and 201 aa in Raman’s model, respectively, UniProt ID: P53178), as we modeled the short *ALG13*-is2 isoform of human Alg13 protein. These two factors can substantially impact the quality of the hAlg13_hm model. Both *Saccharomyces cerevisiae* Alg13 models were obtained as a set of ensembles, so for our analysis we used only the structure of the first ensemble (Figure 3). Additionally, their sequences were identical when compared using BLAST [44]. The yeast Alg13 structures displayed a high overall similarity with two major differences: (i) the α1 helix of Wang’s model was divided into two helices, namely α1 and α2, in Raman’s model, and (ii) the α4 helix in Raman’s model was inserted between two β-strands, namely β4 and β5, while in Wang’s model the β4 and β5 strands were separated only by a short loop. While the yeast Alg13 had a common β-strand pattern (eight mostly parallel β-strands with an antiparallel β4 strand) surrounded by up to nine helices, the hAlg13_hm was relatively smaller. It comprised five parallel β-strands and eight α-helices. It was most similar to Raman’s model with the same pattern of the two consecutive α1 and α2 helices. The 107 residue was located on the loop between the β5 strand and α6 helix.

### 3.5. Analysis of the Mutability of the Asn107 Position c.320A>G p.107N>S

A single-point mutation changing asparagine into serine in position 107 of the human Alg13 is caused by a substitution of the second adenine with guanine in the AAT codon (c.320A>G). According to the results of bioinformatic analyses using the PredictSNP webserver to assess the effect of the Asn107Ser mutation, the mutation is deleterious, although the protein is produced (Supplementary Table S2). However, we could speculate that the introduction of the substitution in the protein structure should display a neutral or stabilizing character. To assess the effect of the mutation on the protein structure and folding, we calculated the differences in the Gibbs free energy of protein folding between the native protein and the mutant variants using the FoldX suite. Interestingly, the introduction of serine was found to have a potentially destabilizing effect for protein folding (1.4 kcal/mol, measured as ΔΔG between the native protein and the mutant). Since the destabilizing effect is small, the protein can still be produced; however, it seems to have an effect on Alg13 function. Given the ambiguous results of transferrin IEF in our patients, we decided to run several experiments and analyze the dynamics of the created model of the human Alg13 structure with and without the UDP-GlcNAc substrate, as well as analyzing the dynamics of the Alg13-Alg14 complex.

### 3.6. Comparison of the Dynamics of Native Alg13 and the Asn107Ser Variant

To investigate the behavior and effect of the introduced mutation on the human Alg13 protein, we ran multiple repetitions of molecular dynamics simulations of the previously built model and the model with the introduced Asn107Ser mutation. To describe the simulations’ results, two commonly used metrics were used, RMSD (Root Mean Square Deviation) and RMSF (Root Mean Square Fluctuation), to measure the quality of each simulation and the model itself. RMSD measures the overall protein stability; it is used to detect conformational changes. RMSF provides information on the average flexibility of a single residue during the simulation time. RMSD and RMSF for the native protein and mutant variant are shown in Supplementary Figure S1. Moreover, since the residue at position 107 is located on a loop, we determined the flexibility of this region by measuring the distance between the Cα atom of the 107 residue and the most stable part of the protein, which was the β4 strand (based on the RMSF values). Higher values suggested a higher flexibility of the region hosting the Asn107Ser mutation, which may be related to problems during substrate transport or complex formation.

Human Alg13 protein is relatively stable. The RMSD plots of the native Alg13 structure fluctuated around 4–5 Å and suggested that the protein does not undergo rapid conformational changes. On the contrary, the RMSD plots of the Asn107Ser variant substantial and rapid conformational changes. In three out of five simulations the RMSD values exceeded 8 Å, while for the two other simulations they were fluctuating near 6 Å (Supplementary Figure S1). However, in one simulation the RMSD values at first reached 10 Å, but after the first 20 ns they decreased near 4 Å and remained on the same level till the end of the simulation. These differences in protein behavior are visible also on the RMSF plots (Supplementary Figure S1). While the RMSF values of the native Alg13 protein share the same pattern in all MD simulations, the RMSF plot of the Asn107Ser variant shows differences between each run. For the native Alg13 protein the most flexible region is its C-terminal with α7 and α8 helices. The same tendency is visible on the RMSF plot of the Asn107Ser variant; however, the RMSF values are higher relative to the RMSF values of the native structure. We also observed that the final positions of residue 107 in both the native and the mutant variant were different from the starting positions.

**Figure 4:**
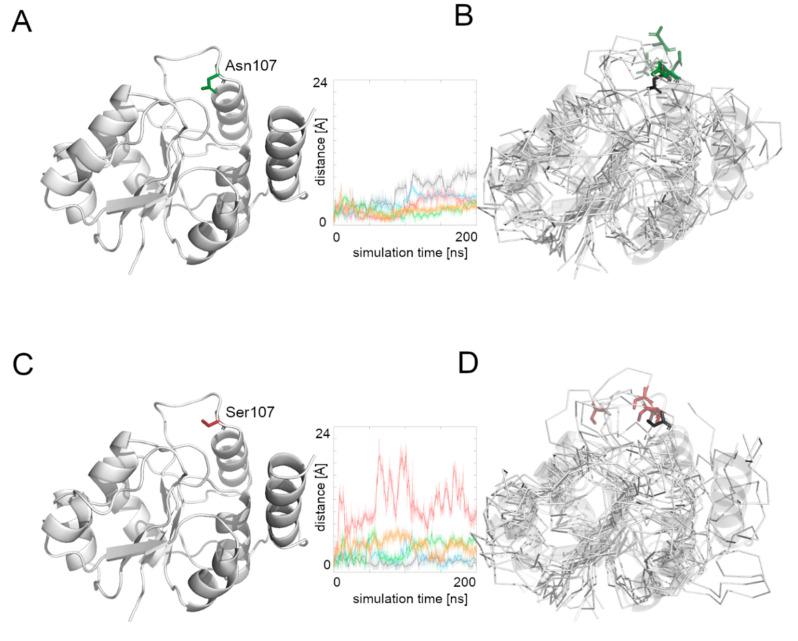
Comparison between the starting point structure of the human Alg13 model and the structures at the end of the simulation. (**A**) Native Alg13 protein structure, (**B**) a set of five structures at the end of the simulation of the native human Alg13, (**C**) Asn107Ser variant of the human Alg13 protein, (**D**) a set of five structures at the end of the simulation of the Asn107Ser variant of the human Alg13. The native Asn107 is colored green, and the Asn107Ser residue is red. The set of five structures at the end of the simulation are shown as ribbons, and the starting point structure (transparent cartoons and black sticks for the starting position of the 107 residue) overlaps with them. Distance plots show the distance between the starting position of 107 residue’s Cα atom and its position in each simulation frame; each colored line represents one simulation.

We then measured the distance between the starting position of residue’s 107 Cα atom and its position during the whole simulation time (Figure 4). The Asn107 residue’s Cα atom was stable for the first half of the most MD simulations, and then it moved away from the starting point position, but the maximal distance did not exceed 10 Å. In the case of the Asn107Ser variant, the Cα atom of the 107 residue was more flexible during the whole MD simulation and in one case moved far away (more than 20 Å) from its starting position. This observation suggests flexibility of the loop on which the residue 107 is located, and also that the Asn107Ser variant shows greater flexibility for this region relative to the native protein. It also suggests that the substrate binding and its further transport may be less effective in the case of the Asn107Ser variant. Therefore, in the next step we focused on substrate transport analysis.

### 3.7. Comparison of the Dynamics of the Native Alg13 and the Asn107Ser Variant in a Complex with UDP-GlcNAc

To investigate the influence of the UDP-GlcNAc substrate on the Alg13 protein behavior, we ran five repetitions of the native Alg13and the Asn107Ser variant complexed with UDP-GlcNAc. The position of the substrate was modeled according to the similar crystal structure of the *P. aeruginosa* MurG protein (PDB ID: 3s2u).

**Figure 5:**
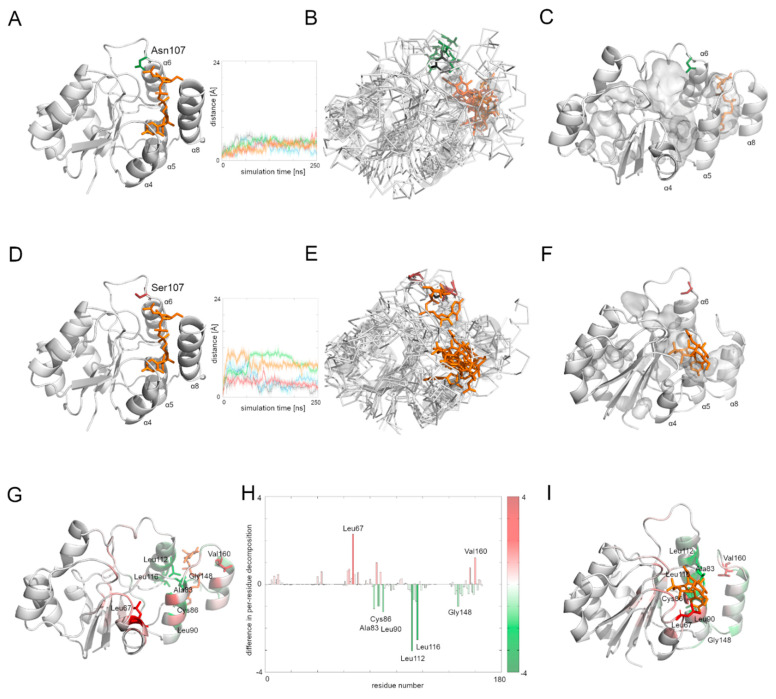
Different interactions with the UDP-GlcNAc substrate of the native Alg13 protein and its Asn107Ser variant. (**A**) Native Alg13 protein structure with substrate, (**B**) a set of five structures at the end of the simulation of the native human Alg13 with substrate, (**C**) structure in the last frame of the simulation of the native Alg13 and position of the substrate, (**D**) Asn107Ser variant of the human Alg13 protein with substrate, (**E**) a set of five structures at the end of the simulation of the Asn107Ser variant of the human Alg13 with substrate, and (**F**) structure in the last frame of the simulation of Asn107Ser variant of the human Alg13 protein and position of the substrate, (**G**) structure in the last frame of the simulation of native Alg13 and residues which interacted differently with the native protein and its Asn107Ser variant, (**H**) differences in per-residue free energy contribution in substrate binding plot, (**I**) structure in the last frame of the simulation of the Asn107Ser variant of the human Alg13 protein. The starting point structure is shown as cartoons with the 107 residue and the substrate shown as sticks. The native Asn107 is colored green, and the Asn107Ser residue is red. The substrate is shown as orange sticks. The set of five structures at the end of the simulation are shown as ribbons, and the starting point structure (transparent cartoons and black sticks for the starting position of the 107 residue) overlaps with them. Distance plots show the distance between the starting position of 107 residue’s Cα atom and its position in each simulation frame; each colored line represents one simulation. For the sample structures of Alg13 with the substrate, cavities are shown using PyMOL software [45] and important secondary structures are marked. The values of the difference in per-residue free energy contribution in the substrate binding were calculated in a way that the energy values of the mutant variant were subtracted from the values of the native protein. The red bars represent regions, which were highly contributing in substrate binding in Asn107Ser variant, but were not identified for the native protein. Residues with the highest differences in per-residue contribution (higher than +1 kcal/mol and lower than −1 kcal/mol) are shown on panels (**G**–**I**).

The human Alg13 protein’s complex with the substrate was relatively stable in both the native protein and the Asn107Ser variant. Interestingly, the RSMD and RMSF plots of the native Alg13 suggest that in the presence of the substrate the protein gained flexibility in the C-terminal and the region comprising the α3 helix and β3 strand (Supplementary Figure S2). On the other hand, the RMSD and RMSF plots of the Asn107Ser variant suggest that its structure became more rigid. The RMSD values for the Asn107Ser variant are lower than those of the native structure, and the same tendency is observed in the case of the RMSF plots. However, in the case of the RMSF plot of the Asn107Ser variant, a high peak is observed in the loop region hosting the mutation that is not present on the RMSF plot of the native protein (Supplementary Figure S2). We measured the distance between the starting position of the Cα atom of the 107 residue during the whole simulation time in both protein-substrate complexes (Figure 5). In the case of the native Alg13 protein in the presence of the UDP-GlcNAc substrate, we observed that the Cα atom moved away in each simulation and the distance fluctuated around 6 Å suggesting that its position was stable. This was not observed in the case of the Asn107Ser variant, in which the distances were greater than in the native protein; in two simulations they exceeded 10 Å. Additionally, when we compared the ending positions of the 107 residue in both Alg13 proteins, we saw that in the case of the native Alg13 protein the position of the 107 residue remained similar in each simulation, while in the case of the Asn107Ser variant the ending positions of the 107 residue were different. Moreover, we observed differences in the UDP-GlcNAc substrate movements. In the case of the native Alg13 the substrate moved into a cavity which was created by a conformational change of α8 helix shifting toward α5 and α6 helices. The UDP-GlcNAc substrate remained in that cavity for most of the simulation time. This behavior of the α5, α6, and α8 helices was not observed in the case of the Asn107Ser variant (Figure 5E,F). It is also worth noting that the α8 helix is thought to be related to the interaction with Alg14 protein [13]. Therefore, our observation might suggest the potential effect of the Asn107Ser variant on Alg13-Alg14 complex formation in the presence of the substrate.

Using MMPBSA and MMGBSA approaches we analyzed the free energy of the substrate binding in both the native and the Asn107Ser variant of the human Alg13 protein (Supplementary Table S3 and Figure S3). The results suggest that in both systems, the binding was stable. We also analyzed the per-residue contribution of the human Alg13 protein with the substrate. While in the case of the native human Alg13 protein only the C-terminal region interacted with the UDP-GlcNAc substrate, in the case of the Asn107Ser variant both the N-terminal and C-terminal, as well as the middle regions of the protein, had contact with the substrate. We also observed that in the complex with the native Alg13 the substrate was located in a cavity created by hydrophobic and negatively charged residues, such as: Leu112 (−3.1 kcal/mol), Leu116 (−2.5 kcal/mol), Leu87 (−1.5 kcal/mol), Leu90 (−1.2 kcal/mol), Ala83 (−1.2 kcal/mol), Cys86 (−1.1 kcal/mol), and Pro147 (−1.0 kcal/mol). We also observed several residues with positive values of per-residue contribution which were repulsing the substrate; however, none of them reached the level of +1 kcal/mol. In the case of the Asn107Ser variant the substrate was positioned differently, and the contributing residues were mostly hydrophobic, polar and positively charged, such as: Leu67 (−2.3 kcal/mol), Val160 (−1.3 kcal/mol), Leu163 (−1.2 kcal/mol), Phe156 (−1.1 kcal/mol), and Ser85 (−1.0 kcal/mol). Similarly, as in the case of the native Alg13 protein, we observed several repulsing residues with a contribution of lower than +1 kcal/mol. We also measured the differences between per-residue contribution between the native protein and the Asn107Ser variant of the human Alg13 protein by subtracting the contribution values of the mutant variant from the native protein values (Figure 5H). The regions on the plot for which the contribution values are positive (shown as red bars) represent the region in which the substrate interacted with the Asn107Ser variant, which shows that in the case of the Asn107Ser variant the substrate binds in different region than in the native protein. Those results suggest that for the Asn107Ser variant the substrate transport is disturbed. In the next step we analyzed the Alg13-Alg14 dimer with the UDP-GlcNAc substrate to gain a whole picture of the substrate transport.

### 3.8. Comparison of the Dynamic of the Native Alg13 and Asn107Ser Variant in the Alg13-Alg14 Complex with UDP-GlcNAc

To examine the behavior of the Alg13-Alg14 dimer, we needed to create another homology model, this time of the dimer. We employed the Modeller 9v19 to create a homology model based on two templates of the MurG protein complexed with the substrate—*E. coli* MurG and *P. aeruginosa* MurG. The position of the substrate was modeled according to the *P. aeruginosa* MurG which had better resolution. The human Alg14 protein structure was downloaded from the SWISS-MODEL repository (ID: Q96F25). This model was also evaluated using ProSA and PROCHECK tools, which assessed its overall quality as good (ProSA Z-score: −5.1, PROCHECK overall score: −0.25). The resulting dimer structure was also assessed using the same tools (ProSA Z-score: −1.42, PROCHECK overall score: −0.15) indicating that the dimer model has good quality; however slightly lower than the quality of Alg13 and Alg14 models separately.

Although the bacterial MurG protein consists of two domains forming a monomer, the human Alg13-Alg14 dimer structurally resembles it. The human Alg14 overlaps with the N-terminal domain, while the Alg13 overlaps with the C-terminal domain. However, the sequence similarity between those proteins is relatively low (below 28% identity). The N-terminal domain of MurG as well as the human Alg14 model, consists of six parallel β-strands surrounded by five α-helices in the case of MurG and seven in the case of the human Alg14 structure. The C-terminal domain of MurG also consists of six parallel β-strands surrounded by seven α-helices and an additional α-helix which interacts with the β-sheet of the N-terminal domain. As previously shown [6], in the Alg13-Alg14 complex the α8 helix of Alg13, which interacts with the Alg14 protein, adopts the same conformation as the last α-helix of the MurG structure.

**Figure 6:**
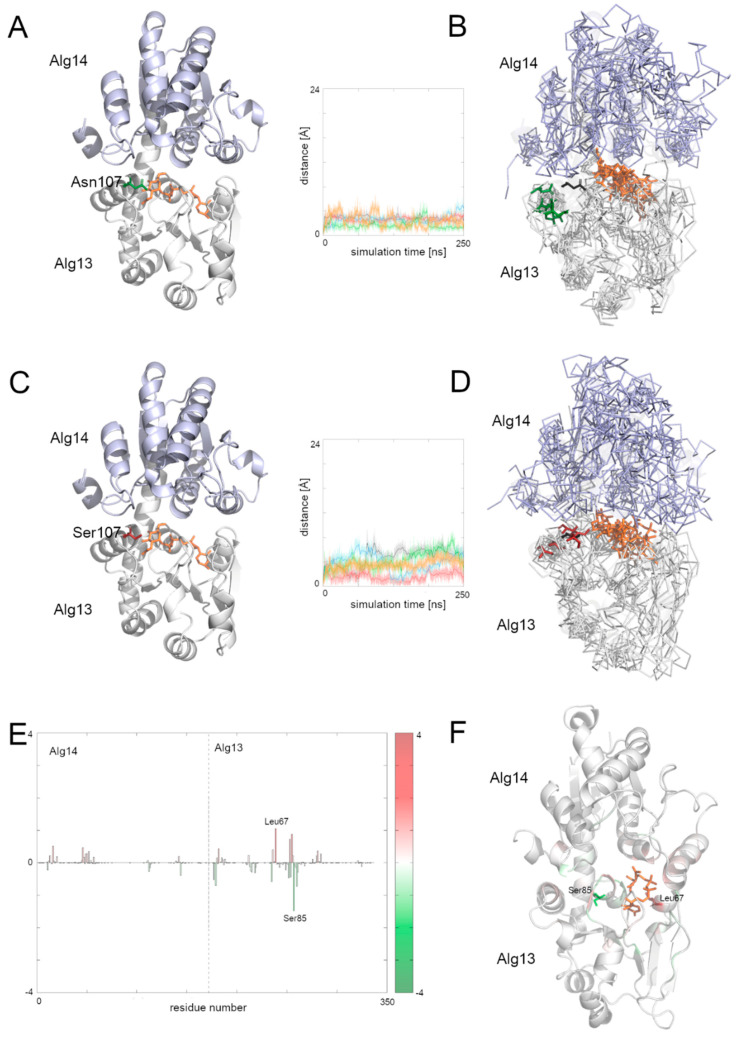
Different interactions with the UDP-GlcNAc substrate of the human Alg13-Alg14 dimer with native Alg13 protein and its Asn107Ser variant. (**A**) Native Alg13-Alg14 dimer structure with substrate, (**B**) a set of five structures at the end of the simulation of the native human Alg13-Alg14 with substrate, (**C**) Alg13-Alg14 dimer with Alg13 Asn107Ser variant with substrate, (**D**) a set of five structures at the end of the simulation of the Alg13-Alg14 dimer with the Alg13 Asn107Ser variant with substrate, (**E**) differences in per-residue free energy contribution in substrate binding plot, (**F**) structure in the last frame of the simulation of the Alg13-Alg14 dimer with the Asn107Ser variant colored according to the differences in per-residue free energy contribution to substrate binding. The starting point structure is shown as cartoons with the 107 residue and the substrate shown as sticks. The Alg14 protein is colored light blue. The protein structure is rotated about 180° with respect to the position of Alg13 in Figure 3. The native Alg13 Asn107 is colored green, and the Asn107Ser residue is red. The substrate is shown as orange sticks. The set of five structures at the end of the simulation are shown as ribbons, and the starting point structure (transparent cartoons and black sticks for the starting position of the 107 residue) overlaps with them. Distance plots show the distance between the starting position of Alg13 107 residue’s Cα atom and its position in each simulation frame; each colored line represents one simulation. The values of the difference in per-residue free energy contribution in the substrate binding were calculated in a way that the energy values of the mutant variant were subtracted from the values of the native protein. Residues with the highest differences in per-residue contribution (higher than +1 kcal/mol and lower than −1 kcal/mol) are shown on panels (**E**,**F**).

To examine the behavior of the system, we analyzed the RMSD of the two domains separately as well as for the whole complex. We also divided the RMSF plot in order to show each domain separately (Supplementary Figure S3). In general, both complexes with the native structure and with the Asn107Ser variant remained stable during the simulation time. Especially the dimer with the native Alg13 domain remained stable, with one exception: in one case the RMSD value increased up to 7 Å after 40 ns and remained at this level till the end of simulation. The RMSF plots of both complexes share mostly the same pattern of high peaks; however the RMSF values differ substantially (Supplementary Figure S4). This suggests that even though the structures are relatively stable, they may display different network of interactions. We therefore also checked the binding free energies of the substrate to the Alg13-Alg14 heterodimer using MMPBSA and MMGBSA (Supplementary Table S4) and the per-residue contribution to the substrate binding (Figure 6 and Supplementary Figure S5). Our results suggest that in both heterodimers (i.e., with the native Alg13 and the Asn107Ser variant) the substrate was stably bound. Supplementary Figure S5 shows that the biggest differences in the contribution to the substrate binding were found in the C-terminal and the middle region of the Alg13 domain. The results show that in the case of the heterodimer with the native Alg13 residues with the highest contribution were hydrophobic and polar, such as: Ser85 of Alg13 (−1.5 kcal/mol), His14 of Alg14 (−1.4 kcal/mol), Glu88 of Alg13 (−1.3 kcal/mol), Thr7 of Alg13 (−1.1 kcal/mol), and Phe5 of Alg13 (−1.1 kcal/mol). On the other hand, the highest contribution in the case of the Alg13-Alg14 dimer with the Asn107Ser variant have the following residues: Leu67 of Alg13 (−1.5 kcal/mol), His14 of Alg14 (−1.4 kcal/mol), Thr16 of Alg14 (−1.3 kcal/mol), Gly13 of Alg14 (−1.2 kcal/mol), Ala83 of Alg13 (−1.1 kcal/mol), and Thr10 of Alg13 (−1.0 kcal/mol). Interestingly, even though the substrate remained in the same cavity in both systems, it interacted with different residues. Only His14 of the Alg14 domain was identified as high contributing in both cases. It is also worth noticing that the Alg13 residue 107 was not involved in the substrate binding. We also compared the distances between the starting position of the Cα atom of the Alg13 107 residue during the whole MD simulation time in both heterodimers (Figure 6). We observed that the overall distance was similar between both heterodimers, which suggests that in the presence of the UDP-GlcNAc substrate and the Alg14 protein, the Alg13 107 residue displayed similar behavior. This also suggests that the Alg13-Alg14 complex may be functional, and that the most crucial step is substrate transport.

## 4. Discussion

Our three reported female patients were diagnosed with ALG13-CDG caused by a single-point mutation c.320A>G, resulting in an Asn107Ser variant of the Alg13 protein. It is one of the most frequently occurring pathogenic variants, but data from the IEF results are still limited. Thus, the two aims of our analyses were: (i) to present the differences in transferrin profiles noted in our patients with ALG13-CDG caused by c.320A>G, who presented with different clinical courses and (ii) to discuss the possible disease mechanism based on protein modeling. The patients’ phenotypic manifestation of the disease varied in regards to epilepsy, the severity of neurodevelopmental delay, and anthropometric parameters. However, as there are no straightforward genotype-phenotype correlations in ALG13-CDG, that was not surprising. Here, we would like to focus on the IEF patterns, which we found to be the most interesting of our results.

### 4.1. IEF of Serum Transferrin

The first description of IEF of serum transferrin was presented in the ALG13-CDG patient described by Smith-Packard et al. [2]. It showed a normal pattern. Before this publication appeared, none of the female patients reported in the literature with the same pathogenic variant had glycosylation studies carried out. Later, in 2016, Western blot analysis of a 6-year-old female patient who developed infantile spasms showed the normal glycosylated transferrin isoforms [46]. In the most recent publication by Alsharhan et al. [7], transferrin IEF was performed for only two patients out of 11 ALG13-CDG individuals, and gave normal results. Additionally, an analysis of glycans by MS was presented, revealing abnormalities in nine patients, but transferrin IEF was not performed. Referring to our results, which showed the differences in the IEF pattern among three patients, it can be supposed that it is a result of several factors: the patients’ age (ranging from 2 1/12 years for Patient 2 to 5 10/12 years for Patient 3 at the moment of the analysis), the clinical severity (the mildest spectrum for Patient 1, the most severe for Patient 3), or even antiepileptic drugs (see [10]).

Transferrin IEF is one of the three methods (together with CE and HPLC) currently used worldwide for CDG screening (in more or less equal amounts), according to the Congenital Disorders of Glycosylation Final Report 2020 [47]. There are also other methods available, such as matrix-assisted laser desorption ionization—time-of-flight (MALDI TOF) or quadrupole time-of-flight (QTOF) mass spectrometry. However, at present, these methods are available within a limited range of equipment, and the analyses are much more expensive than using transferrin IEF, CE, or HPLC [48]. We are aware of the limitation of the presented data (for instance, it is important to complete each patient’s diagnosis with MS methods). However, the presented results of transferrin isoform analysis (regardless of whether IEF or CE was used) give a rationale for further research. Moreover, our data suggest the necessity for further studies to verify the differences and specificity in the IEF patterns for c.320A>G ALG13-CDG.

### 4.2. Implications from Alg13 Modeling 

A single change in the nucleotide sequence can have various effects; it can affect the protein stability, folding, substrate binding, or (due to in interaction with other proteins) it can also be lethal. Here, a single-point mutation c.320A>G of the human *ALG13* gene results in the Alg13 Asn107Ser variant. The bioinformatic analysis of the Asn107Ser mutation suggested the deleterious character of the amino acid replacement, according to seven out of eight tools of the PredictSNP server (see Supplementary Table S2). On the other hand, the calculated effect of the Asn107Ser variant was shown to have a neutral or slightly destabilizing character (Supplementary Figure S1). The mutation is reported to be a spontaneous, de novo mutation, the effect of which on the protein mechanism remains unknown. Here, we discuss the plausible effect of the Asn107Ser variant on the N-glycosylation process.

We used a homology model built by the I-TASSER webserver based on several available templates, such as the structures of yeast Alg13. Human *ALG13* has two isoforms, long *ALG13-is1* and short *ALG13-is2*, which share the N-terminal region (125 amino acids) that hosts the catalytic domain, and the isoforms differ substantially in the C-terminal region. The C-terminal region of the long *ALG13-is1* was shown to have ubiquitinase activity, while the C-terminal region of the short *ALG13-is2* is thought to be important in shuttling the Alg13 protein to the ER [8]. Moreover, the short *ALG13-is2* isoform was analyzed by Gao et al., who showed that the C-terminal region of the Alg13 protein is crucial for the interaction with the Alg14 protein [13,49]. Since the structure of human Alg14 has not yet been experimentally solved, and no template from a closely related organism is available. In our study, we used a model created by SWISS-MODEL and deposited in their database repository. Recently, another structure prediction method has been added into the SWISS-MODEL repository. AlphaFold, the most accurate template-free method [50], predicted an Alg14 structure similar to the model created by SWISS-MODEL ([AF-Q96F25-F1 model]). However, unlike the SWISS-MODEL, the AlphaFold prediction comprised an N-terminal α-helix, which acts as a membrane anchor.

In our study, we used three models obtained by different structure prediction methods: hAlg13_hm was predicted by I-TASSER, Alg14 model by SWISS-MODEL, and the dimer model by Modeller. All those tools are template-based, so the accuracy of the predicted model relies strongly on the quality and sequence similarity between the target and template models. To assess the quality of these models we used two different approaches which indicated good quality of the models. It is worth noting that the dimeric structure of the Alg13-Alg14 complex for modeling of which we used other homology models was less accurate. However, as shown in Supplementary Figures S1–S3, all models, except the apo structure of the Asn107Ser variant, kept their overall stability and did not undergo dramatic conformational changes.

Both the Alg13 and Alg14 proteins consist of long loops that connect the secondary structure elements with each other. Such long loops may affect the RMSD and RMSF plots, which we used for the description and evaluation of the analyzed models, since they introduce flexibility. It must be kept in mind that a protein structure requires some degree of flexibility, which allows it to change its conformation in order to interact with substrates, products, and/or other proteins. Therefore, we compared the RMSD plots with those presented by Saxena et al. [51], who presented novel *Mycobacterium tuberculosis* MurG inhibitors. They showed that during the molecular dynamics, simulations of the apo structure of MurG, the RMSD values fluctuated near 4–6 Å, which indicated that our Alg13 model is relatively stable.

In our study, we ran five MD simulations of the apo structure of human Alg13 protein as well as it complex with the UDP-GlcNAc substrate, for both native Alg13 and the Asn107Ser variant. The homologous C-terminal region of MurG, which is called the α/β/α motif, comprises a large part of the donor binding site [52]. It is worth noting that a similar α/β/α motif hosts the Asn107Ser substitution in Alg13. We measured the distance between the starting point position of the Cα atom of the 107 residue and its position during whole MD simulation time. In the case of the apo structure of the human Alg13 protein, we observed that the native structure was rigid, while the Asn107Ser variant was highly flexible. This tendency was also observed in the case of the Alg13 protein complexed with the UDP-GlcNAc substrate; however, the native Alg13 protein gained flexibility in its C-terminal region and the loop region in which the residue 107 is located. The Asn107Ser variant in the presence of the substrate was more rigid than the apo structure; however, it was still more flexible than the native Alg13 with substrate. Interestingly, we also observed that in the presence of the substrate, the native Alg13 undergoes a conformational rearrangement of its C-terminal region, during which the α8 helix moves closer to the α5 and α6 helices creating an additional cavity in which the substrate entered, staying there for most of the simulation time (Figure 5C,F). This behavior was not observed during simulations of the Asn107Ser variant; the substrate remained in the binding pocket. Those observations were confirmed by the per-residue decomposition of the substrate binding analysis for both native and the Asn107Ser variant of the human Alg13 with bound substrate. We suggest that such structural rearrangement may be crucial for the substrate transport and further complex formation with the Alg14 protein. Based on those results, we can suggest that in the case of Asn107Ser variant the substrate transport is insufficient. However, without knowledge of the rate limiting step, we cannot speculate whether the lack of sufficiency is related to product association or its dissociation. Additionally, given the fact that the C-terminal helix region is crucial for the Alg13-Alg14 complex formation, our observation may suggest that such a conformational change in the presence of the substrate may be important and can disturb the Alg13-Alg14 complex formation.

Finally, we built a homology model of the Alg13-Alg14 heterodimer, using two currently available structures with bound substrate: *E. coli* MurG (PDB ID: 1nlm) and *P. aeruginosa* MurG (PDB ID: 3s2u). Gao et al., who showed that the Alg13 and Alg14 proteins are homologous to the bacterial MurG (N-terminal corresponds to Alg14, while the C-terminal corresponds to Alg13 [13,49]), also created a model obtained by a combination of the solved structure of yeast Alg13 with a homology model of Alg14 [49]. They reoriented the C-terminal α8 helix of Alg13 based on the MurG structure to reassemble the Alg13-Alg14 complex. They also claimed that the assembly of Alg13 and Alg14 subunits is critical for the activity of N-acetylglucosamine transferase, although little is known about its mechanism.

The results of the Alg13-Alg14 dimer simulations showed a different tendency regarding the movement of the Cα atom of residue 107 of human Alg13. When we simulated only the Alg13 protein, for both the apo structure and the complex with the substrate, we observed a higher flexibility of the Asn107Ser variant in the region hosting the mutation. However, during simulations of the dimer structure, the flexibility of both dimer structures was similar. The substrate, during simulations of both dimers, comprising the native and Asn107Ser variant of Alg13, remained in the binding pocket. Therefore, we speculate that the overall Alg13-Alg14 complex is functional, and that the Asn107Ser variant disturbs the substrate transport from Alg13 with bound substrate to the Alg13-Alg14 heterodimer. Increased flexibility of the native Asn107 residue relative to the simulations of the monomeric Alg13 may suggest its role during the catalytic reaction. Our results also confirmed the findings of Ng et al. [6], who identified patients with ALG13-CDG with the most common Asn107Ser mutation, as well as another previously unknown variant, Ala81Thr. They superimposed their model of human Alg13 with a crystal structure of *P. aeruginosa* MurG with bound UDP-GlcNAc (PDB ID: 3s2u) and concluded that both Asn107 and Ala81 residues are located in a region that is involved in substrate binding. Therefore, we suspect that despite the fact that the results of the MD simulations of both dimers suggested that the binding of the substrate is similar, the Asn107Ser variant of the human Alg13 may cause issues related to the transport of the substrate.

## 5. Conclusions

A single-point mutation c.320A>G in the *ALG13* gene results in the Asn107Ser variant of the Alg13 protein. According to the bioinformatic analyses, the mutation itself is not deleterious; however, it causes a multisystemic metabolic disorder, called ALG13-CDG, in the patients with the c.320A>G mutation.

Analysis of five repetitions of MD simulations of the homology model of the human Alg13, both as an apo structure or in complex with the UDP-GlcNAc substrate suggests that the Asn107Ser variant alters flexibility of the loop hosting this mutation. Such influence was not observed for the Alg13-Alg14 dimer. The observations from the MD simulations, as well as data from a thorough literature search allowed us to speculate about a plausible mechanism for the role of the Asn107Ser variant of the Alg13 protein during N-glycosylation. While Alg14 is membrane-bound, Alg13 acts as a transporter, it binds the substrate and moves it to Alg14. In the presence of the substrate, Alg13 and Alg14 form a heterodimer in which the second step of the N-glycosylation occurs. Our results suggest that in the case of the Asn107Ser variant of the human Alg13 protein, the Alg13-Alg14 complex remains functional, but the process of the substrate binding to Alg13 and its further transport might be disturbed, thus disturbing the N-glycosylation process.

We also showed the IEF results of three female patients diagnosed with ALG13-CDG caused by c.320A>G. Since the ALG13-CDG caused by c.320A>G is very rare, we would like to report on further female patients with different spectra of clinical severity, with abnormal patterns of serum transferrin.

## Figures and Tables

**Table 1:** Isoelectric focusing (IEF) of transferrin in our patients with ALG13 c.320A>G variant (CDT values exceeding the reference range are marked with red). The letter below the patient’s number corresponds to the band showed in Figure 2.

	Reference Range %	Patient 1C	Patient 1	Patient 2D	Patient 2	Patient 3 A	Patient 3 B
Asialo-	0	0	0	**0.5**	0	0	0
Monosialo-	0	0	0	**1.4**	0	0	0
Disialo-	1.5–6.2	4.5	3.9	**7.0**	5.0	5.3	**6.3**
Trisialo-	7.4–17.1	15.4	14.9	8.6	10.2	11.1	12.5
Tetrasialo-	55.7–67.2	56.2	56.7	55.0	59.7	50.9	55.8
Pentasialo-	13.2–19.9	21.5	20.0	23.6	21.6	27.3	21.9
Hexasialo-	2.5–5.6	2.5	4.5	3.9	3.5	5.4	3.5

**Table 2:** Levels of transferrin in our patients with *ALG13* c.320A>G variant and abnormal IEF pattern (values exceeding the reference range are marked with red).

Reference Range [%]	≤1.3
Patient 1	0.4
Patient 2	**>2.1**
Patient 3	**>1.8**

## Data Availability

Molecular dynamics simulations data available on request due to restrictions related to the size of the files.

## Supplementary Materials

The following supporting information can be downloaded at: https://www.mdpi.com/article/10.3390/biom12030398/s1, Table S1: Anthropometric measurements; Table S2: Results of the assessment of the Asn107Ser mutation examined by PredictSNP webserver and other tools; Figure S1: Comparison of RMSD and RMSF plots of the native Alg13 and the Asn107Ser variant; Figure S2: Comparison of RMSD and RMSF plots of the native Alg13 and the Asn107Ser variant complexed with the UDP-GlcNAc substrate; Table S3: Results of the MMPBSA and MMGBSA assessments of the stability of the Alg13 complex with the UDP-GlcNAc substrate; Figure S3: Differences in the per-residue decomposition between the native and the Asn107Ser variant of the human Alg13 protein with the UDP-GlcNAc substrate; Figure S4: Comparison of RMSD and RMSF plots of the Alg13-Alg14 dimer consisting of native human Alg13 and the Asn107Ser variant of the human Alg13 complexed with the substrate; Table S4: Results of the MMGBSA and MMPBSA assessments of the stability of the Alg13-Alg14 complex with the substrate; Figure S5: Differences in the per-residue decomposition between the Alg13-Alg14 heterodimer with the native and the Asn107Ser variant of the human Alg13 protein with the UDP-GlcNAc substrate.
