# Supplementary material for: Structural Analysis of the Effect of Asn107Ser Mutation on Alg13 Activity and Alg13-Alg14 Complex Formation and Expanding the Phenotypic Variability of ALG13-CDG
Source: Biomolecules. 2022 Mar 4;12(3):398. doi: 10.3390/biom12030398 (PMC8945535; doi:10.3390/biom12030398)

biomolecules

MDPI

Structural analysis of the effect of Asn107Ser mutation on Alg13 activity and Alg13-Alg14 complex formation and expanding the phenotypic variability of ALG13-CDG

Karolina Mitusińska, Artur Góra, Anna Bogdańska, Agnieszka Rożdżyńska-Świątkowska, Anna Tylki-Szymańska, and Aleksandra Jezela-Stanek

Supplementary Data

Supplementary Data include five figures and two tables.

Supplementary Table S1. Anthropometric measurements.
Supplementary Table S2. Results of the assessment of the Asn107Ser mutation examined by PredictSNP webserver.
Supplementary Figure S1. Comparison of RMSD and RMSF plots of native Alg13 and the Asn107Ser variant.
Supplementary Figure S2. Comparison of RMSD and RMSF plots of native Alg13 and the Asn107Ser variant complexed with the UDP-GlcNAc substrate.
Supplementary Table S3. Results of the MMPBSA and MMGBSA assessments of the stability of the Alg13 complex with the UDP-GlcNAc substrate.
Supplementary Figure S3. Differences in the per-residue decomposition between the native and the Asn107Ser variant of the human Alg13 protein with the UDP-GlcNAc substrate.
Supplementary Figure S4. Comparison of RMSD and RMSF plots of the Alg13-Alg14 dimer consisting of native human Alg13 and the Asn107Ser variant of the human Alg13 complexed with the UDP-GlcNAc substrate.
Supplementary Table S4. Results of the MMGBSA and MMPBSA assessments of the stability of the Alg13-Alg14 complex with the UDP-GlcNAc substrate.
Supplementary Figure S5. Differeces in the per-residue decomposition between the Alg13-Alg14 heterodimer with the native and the Asn107Ser variant of the human Alg13 protein with the UDP-GlcNAc substrate.

Supplementary Table S1. Anthropometric measurements.

**craniofacial (cm)**		
head circumference	OFC	maximum circumference in frankfurt plane at level of glabella and opisthocranion
**craniofacial (mm)**		
head length	g-op	glabella to opisthocranion
head breadth	eu-eu	eurion to eurion
forehead breadth	ft-ft	frontotemporale to frontotemporale
**somatometric (cm)**		
body height		basis to vertex
head and neck length (v-sst)	v-sst	vertex to suprasternale
lower extremity length (B-sy)	B-sy	basis to symphysion
trunk length (sst-sy)	sst-sy	suprasternale to syphysion
upper extremity length (a-daIII)	a-daIII	acromion-dactylion III
biacromial diameter (a-a)	a-a	acromion-acromion
chest width (thl-thl)	thl-thl	thoracolaterale-thoracolaterale
chest sagittal diameter (xi-ths)	xi-ths	xiphoid-thoracic spinal
hip width (ic-ic)	ic-ic	iliocristale to iliocristale
chest circumference		circumference in horizontal plane at level of xiphoid

Supplementary Table S2. Results of the assessment of the effect of Asn107Ser mutation examined by PredictSNP webserver. PredictSNP gathers results from different tools, such as MAPP, PolyPhen-1, PolyPhen-2, etc, for evaluation of particular mutation. Finally, it provides predictions of individual tools and a consensus prediction by itself. Table shows the confidence of the predictions and the predicted effect of the Asn107Ser mutation.

	confidence	effect
PredictSNP	87%	deleterious
MAPP	76%	deleterious
PhD-SNP	77%	deleterious
PolyPhen-1	59%	deleterious
PolyPhen-2	68%	deleterious
SNAP	79%	deleterious
SIFT	72%	deleterious
nsSNPAnalyzer	-	unknown
PANTHER	65%	deleterious

Supplementary Figure S1. Comparison of heavy atoms RMSD and Cα atoms RMSF plots of the native Alg13 and the Asn107Ser variant. A) RMSD plot of the native human Alg13 protein and B) the Asn107Ser variant, C) RMSF plot of the native human Alg13 protein and D) the Asn107Ser variant. Each colored line represents each repetition of molecular dynamics simulation.

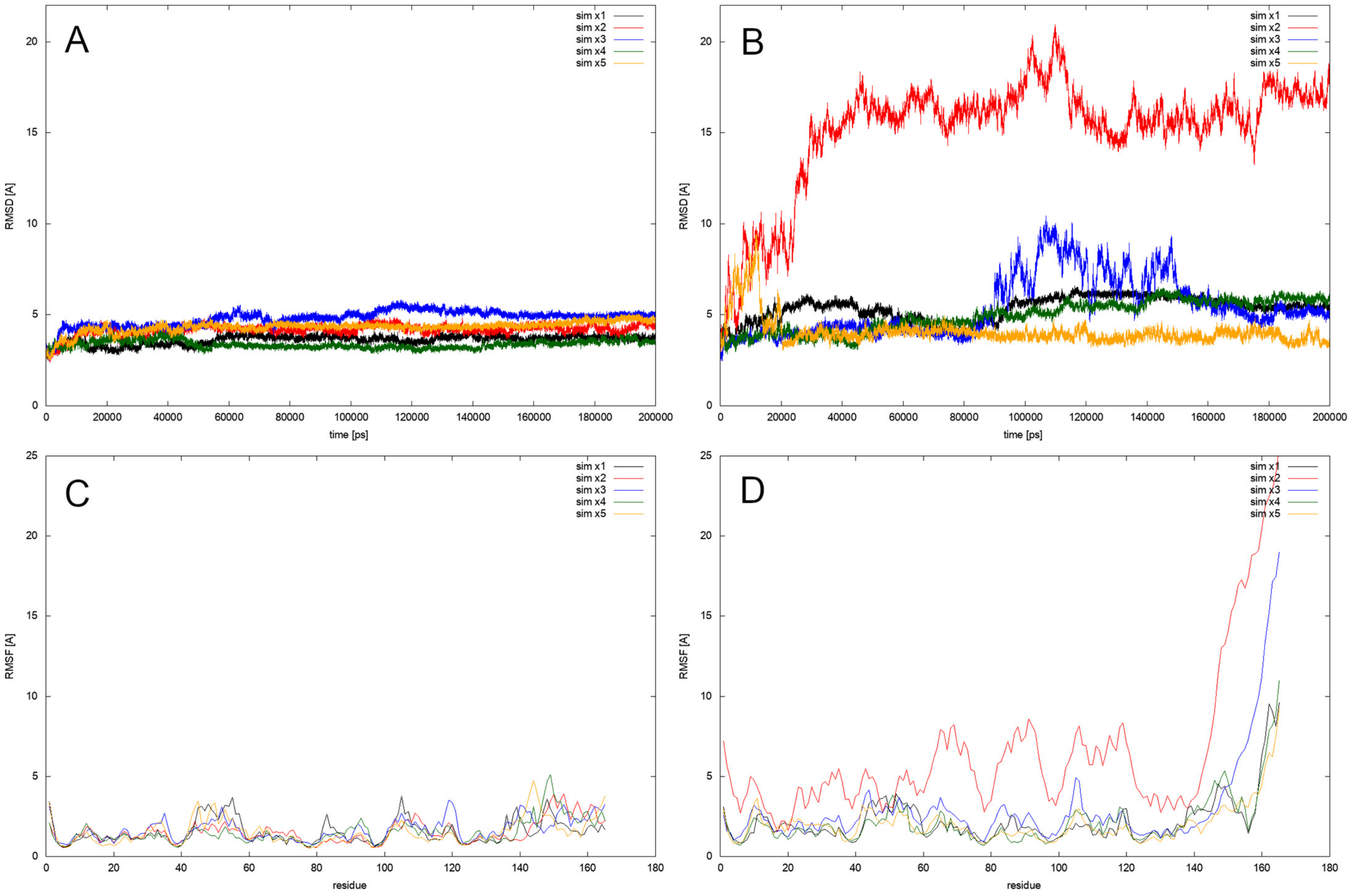

Supplementary Figure S2. Comparison of heavy atoms RMSD and Cα atoms RMSF plots of the native human Alg13 and the Asn107Ser variant in a complex with the UDP-GlcNAc substrate. A) RMSD plot of the native human Alg13 protein and B) the Asn107Ser variant, C) RMSF plot of the native human Alg13 protein and D) the Asn107Ser variant. Each colored line represents each repetition of molecular dynamics simulation.

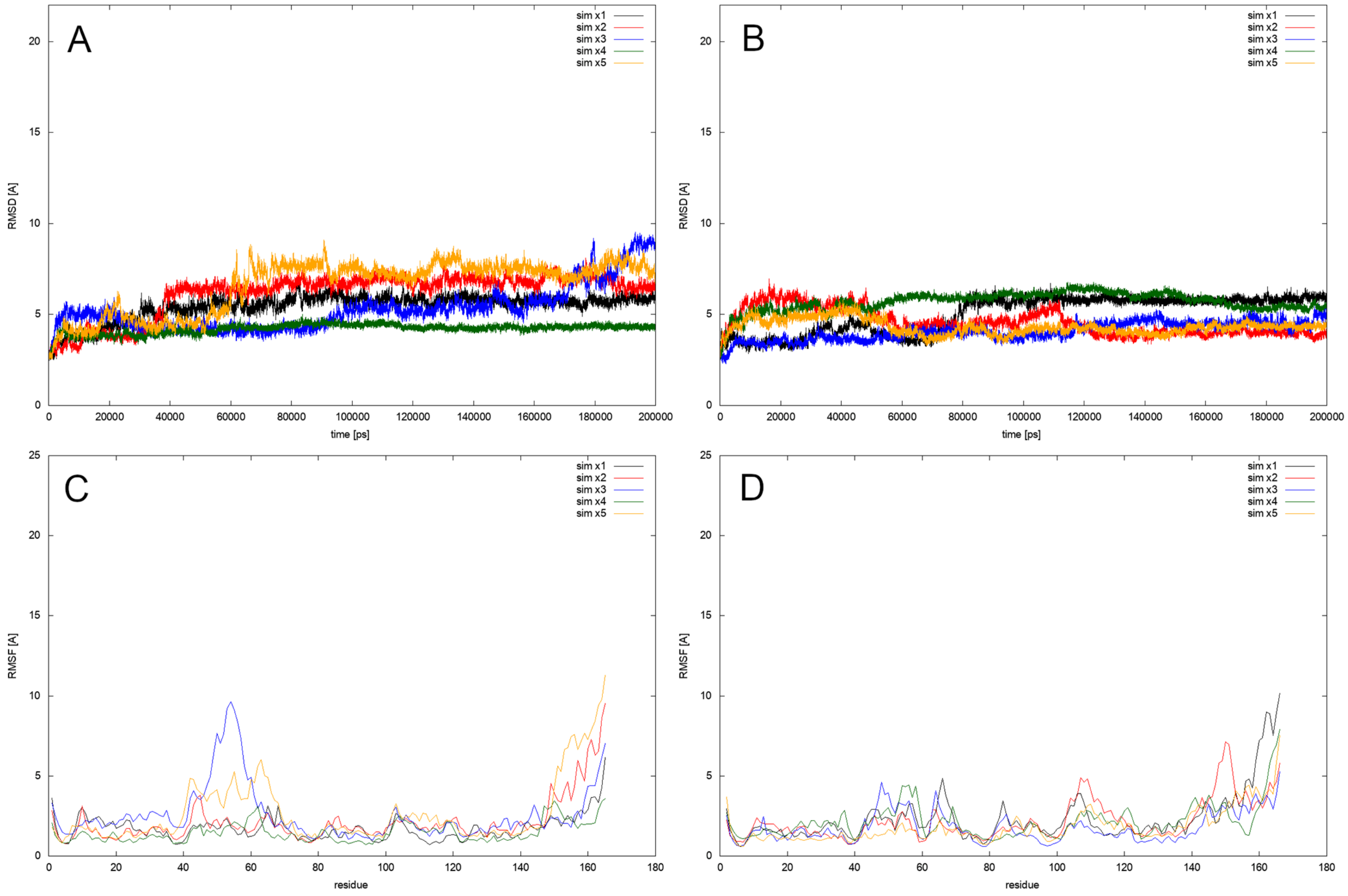

Supplementary Table S3. Results of the MMPBSA and MMGBSA assessments of the stability of the Alg13 complex with the UDP-GlcNAc substrate.

	MMGBSA	MMPBSA
Native Alg13	-59.9 ± 10.9	-24.4 ± 6.6
Asn107Ser	-51.8 ± 8.0	-14.6 ± 5.6

Supplementary Figure S3. Differences in the per-residue decomposition between the native and the Asn107Ser variant of the human Alg13 protein with the UDP-GlcNAc substrate. A) Per-residue free energy contribution to the substrate binding plot for the native Alg13 protein in complex with the UDP-GlcNAc substrate. B) Native Alg13 structure in complex with the substrate (last frame of the first repetition). C) Per-residue free energy contribution to the substrate binding of the Asn107Ser variant of the human Alg13 protein D) Asn107Ser variant in complex with the substrate (last frame of the last simulation). Protein structures are shown as cartoon and colored according to the respective per-residue free energy contribution plot, the GlcNAc substrate is shown as orange sticks.

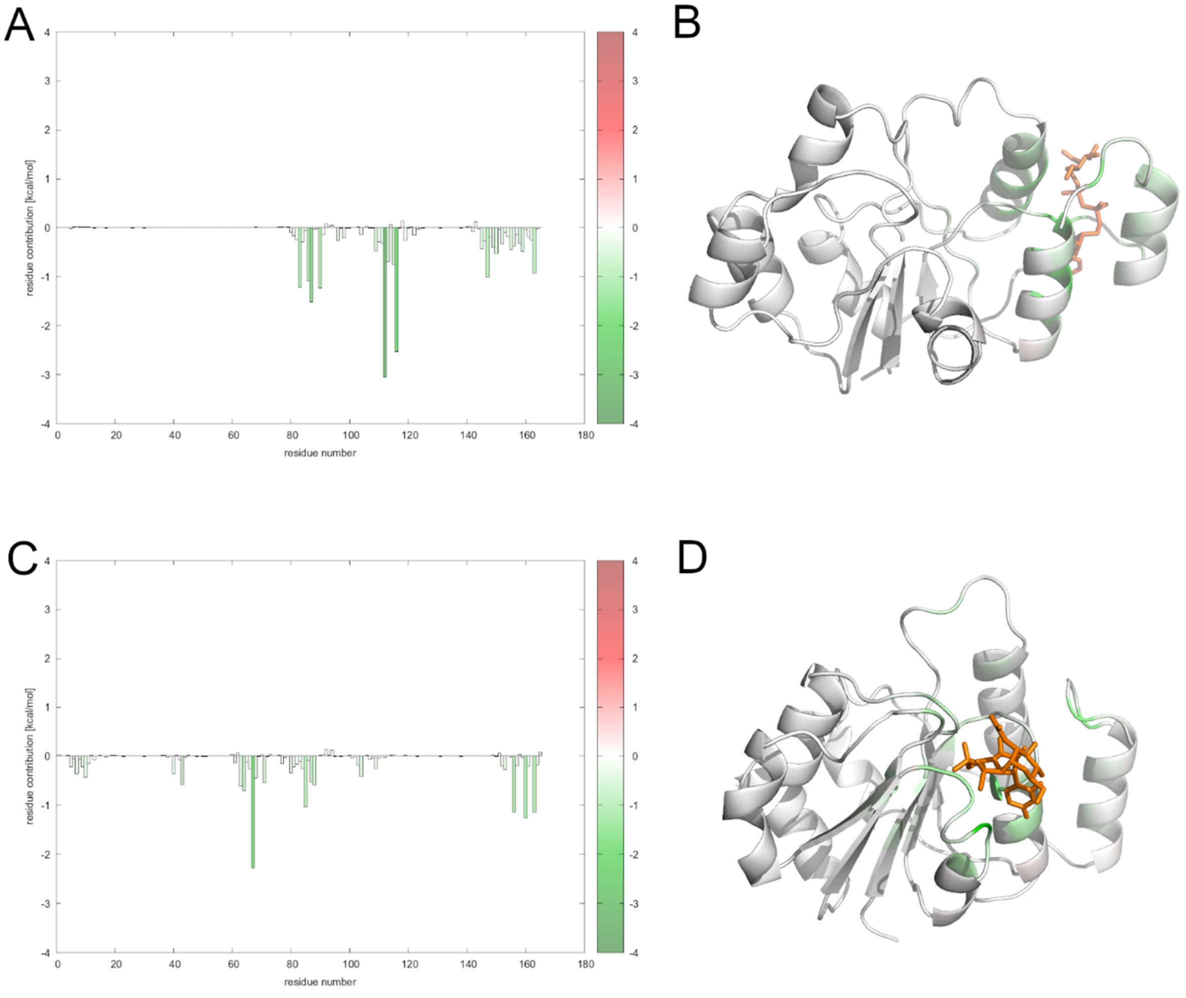

Supplementary Figure S4. Comparison of heavy atoms RMSD and Cα atoms RMSF plots of the Alg14-Alg13 dimer consisting of the native human Alg13 and the Asn107Ser variant of human Alg13 in complex with the GlcNAc substrate. A) RMSD plot of the Alg14-Alg13 dimer consisting of the native human Alg13 protein and B) the Asn107Ser variant, C) RMSD plot of the native Alg13 domain and D) the Asn107Ser variant of the Alg13 protein, E) RMSD plot of the Alg14 domain creating a dimer with the native Alg13 protein, F) RMSD plot of the Alg14 domain creating a dimer with the Asn107Ser variant of the Alg13 protein, G) RMSF plot of the Alg14-Alg13 dimer consisting of the native human Alg13 protein and H) the Asn107Ser variant. Each colored line represents each repetition of molecular dynamics simulation.

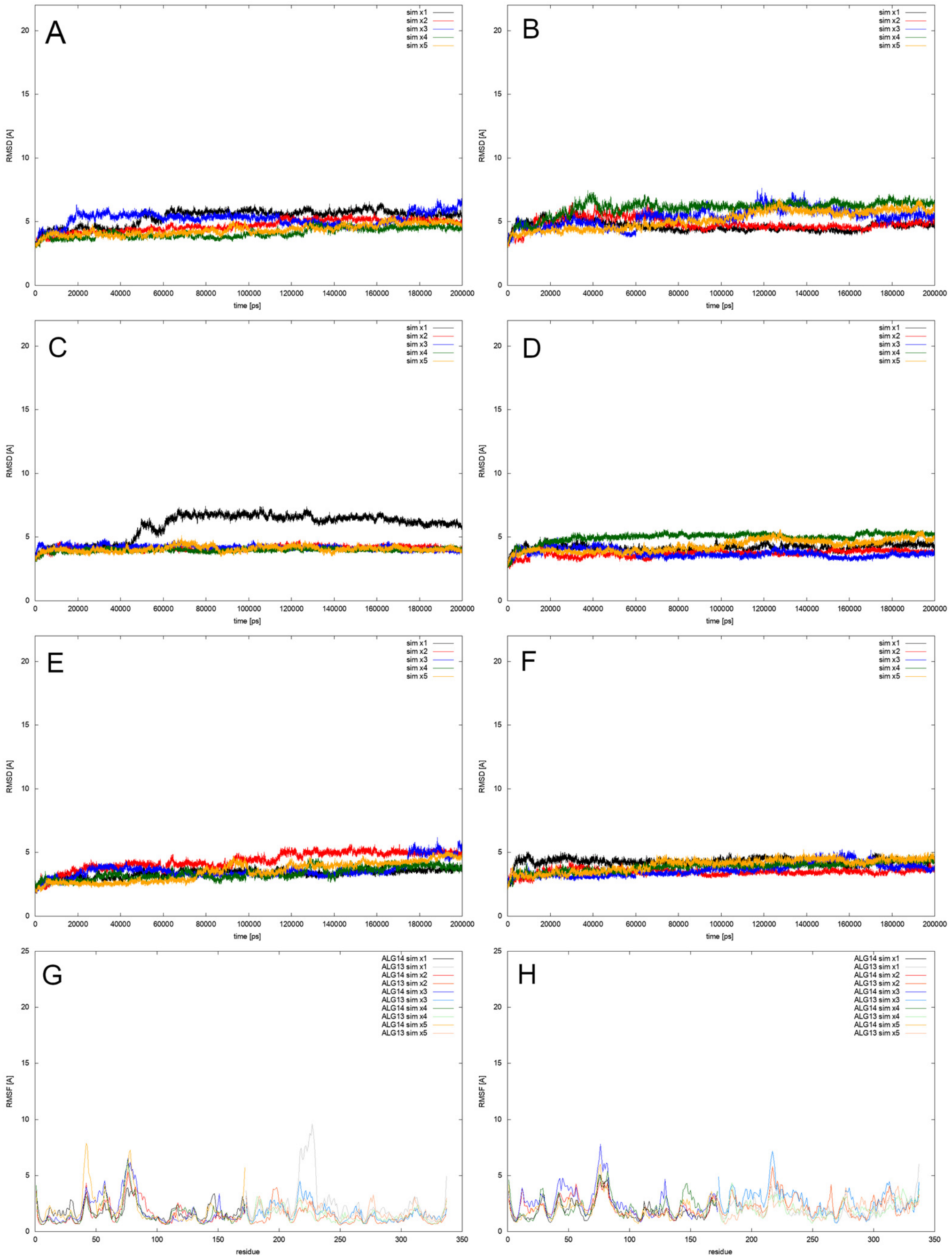

Supplementary Table S4. Results of the MMPBSA and MMGBSA assessments of the stability of the Alg13-Alg14 complex with the substrate.

	MMGBSA	MMPBSA
Native Alg13	-59.4 ± 6.6	-6.6 ± 8.5
Asn107Ser	-58.6 ± 4.6	-7.0 ± 7.2

Supplementary Figure S5. Differences in the per-residue decomposition between the Alg13-Alg14 heterodimer consisting of native and the Asn107Ser variant of the human Alg13 protein with the UDP-GlcNAc substrate. A) Per-residue free energy contribution to the substrate binding plot for the Alg13-Alg14 heterodimer comprising of native Alg13 protein in complex with the UDP-GlcNAc substrate. B) Alg13-Alg14 structure consisting of the native Alg13 in complex with the substrate (last frame of the first repetition). C) Per-residue free energy contribution to the substrate binding plot for the Alg13-Alg14 heterodimer comprising of the Asn107Ser variant of the Alg13 protein in complex with the UDP-GlcNAc substrate. D) Alg13-Alg14 structure consisting of the Asn107Ser variant of the Alg13 in complex with the substrate (last frame of the first repetition). Protein structures are shown as cartoon and colored according to the respective per-residue free energy contribution plot, the GlcNAc substrate is shown as orange sticks.

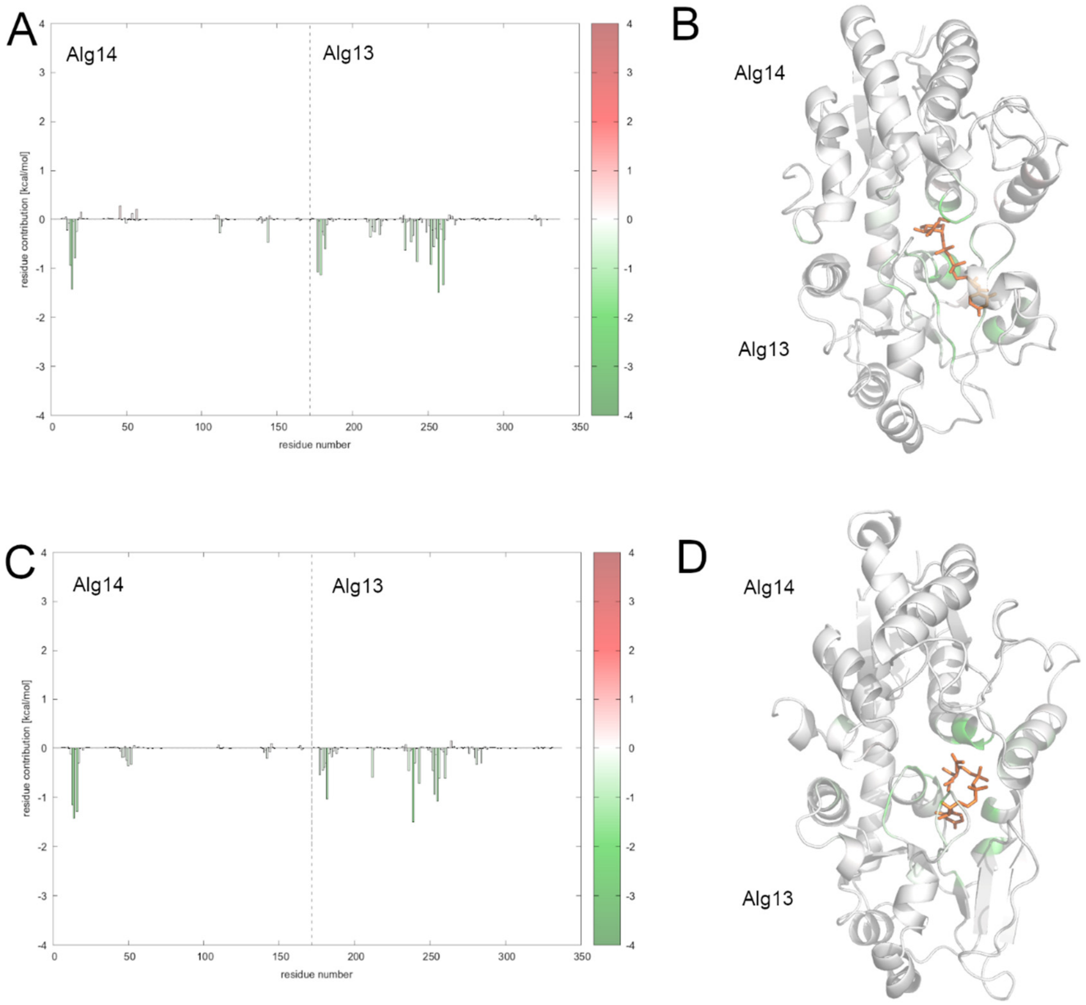

Supplement: Supplementary file 1 [file biomolecules-12-00398-s001.zip › biomolecules-1617837-supplementary.pdf]
